# Patients with substance use disorder who have higher alexithymia levels present more suicidality history: Preliminary results in an outpatient addiction treatment center in Spain

**DOI:** 10.1192/j.eurpsy.2021.1552

**Published:** 2021-08-13

**Authors:** R. Palma Álvarez, C. Daigre, E. Ros-Cucurull, P. Serrano-Pérez, G. Ortega-Hernandez, M. Perea-Ortueta, J. Vendrell-Serres, L. Gallego, J.A. Ramos-Quiroga, L. Grau-López, C. Roncero

**Affiliations:** 1 Psychiatry, Hospital Universitari Vall d’Hebron, Barcelona, Spain; 2 Psychiatry, Complejo Asistencial Universitario de Salamanca. Instituto de Biomedicina de Salamanca., Salamanca, Spain

**Keywords:** alexithymia, Substance Use Disorder, Suicidal ideation, Suicide attempts

## Abstract

**Introduction:**

Patients with substance use disorders (SUD) have higher alexithymia levels and present frequently suicidal ideation (SI) and suicide (SA) [1,2]. Beside, alexithymia has been related to suicidal behaviors in several psychiatric disorders[3]. Although, there are some studies on alexithymia and suicidality in SUD patients, to our knowledge there are no studies on this issue in Spanish population.

**Objectives:**

To compare the alexithymia levels in SUD patients with and without SI and SA in an outpatient addiction treatment center in Spain.

**Methods:**

This is a cross-sectional study performed on 110 patients (74.3%males; mean age 43.6±14.5years old) for whom we had information from the Toronto Alexithymia Scale(TAS-20) and the presence or not of lifetime SI and SA.

**Results:**

Lifetime SI and SA were present in 55.5% and 35.5% of the sample respectively. The mean score of TAS-20, difficulties identifying feelings (DIF), difficulties describing feelings (DDT), and externally-oriented thinking(EOT) were 57.2±13.3, 20.0±7.0, 14.7±4.5, and 22.5±4.5 respectively.

**Figure:**
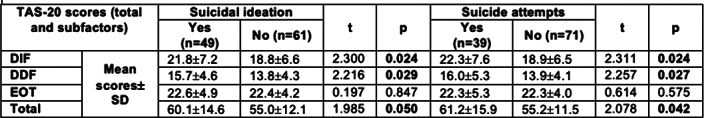

**Conclusions:**

SI and SA may be related to alexithymia levels. Hence, alexithymia should be further analyzed in SUD patients in longitudinal studies in order to analyze the bilateral association with suicidal spectrum behaviors. REFERENCES Rodríguez-Cintas L, et al. Factors associated with lifetime suicidal ideation and suicide attempts in outpatients with substance use disorders. Psychiatry Res. 2018;262:440-5. Morie KP, et al. Alexithymia and Addiction: A Review and Preliminary Data Suggesting Neurobiological Links to Reward/Loss Processing. Curr Addict Rep. 2016;3(2):239-48. Hemming L, et al. A systematic review and meta-analysis of the association between alexithymia and suicide ideation and behaviour. J Affect Disord. 2019;254:34-48.

